# Multicenter experience of pipeline embolization device used in small caliber vessels (< 2 mm) for intracranial aneurysm treatment and mid-term results

**DOI:** 10.3389/fneur.2026.1778770

**Published:** 2026-04-08

**Authors:** Huijian Ge, Siming Gui, Jia Jiang, Yang Zhao, Xiheng Chen, Sheng Bi, Lu Zheng, Jiahe Wei, Youxiang Li

**Affiliations:** 1 Department of Neurosurgery, Beijing Tiantan Hospital and Beijing Neurosurgical Institute, Capital Medical University, Beijing, China; 2 Department of Neurosurgery, Peking Union Medical College Hospital, Peking Union Medical College, Chinese Academy of Medical Sciences, Beijing, China; 3 Department of Neurosurgery, Beijing Chaoyang Hospital, Capital Medical University, Beijing, China; 4 School of Basic Medical Sciences, Capital Medical University, Beijing, China

**Keywords:** aneurysm occlusion, flow diverter, in-stent stenosis, intracranial aneurysm, ischemic complications, small vessel

## Abstract

**Background:**

Flow diversion stents (FDS), particularly the Pipeline Embolization Device (PED), have been widely used for intracranial aneurysm (IA) treatment. However, data on PED deployment in small-caliber parent vessels (< 2 mm) remain limited. This study aimed to evaluate the technical feasibility, safety, and efficacy of PED for IAs arising from small-caliber vessels.

**Methods:**

This was a multicenter retrospective study enrolling 71 eligible patients with IA located in parent vessels < 2 mm who underwent PED implantation. The primary safety endpoint included procedure-related death, symptomatic stroke, intracranial hemorrhage and asymptomatic in-stent stenosis; the primary efficacy endpoint was complete aneurysm occlusion [O’Kelly-Marotta (OKM) grade D] on follow-up digital subtraction angiography (DSA). Binary logistic regression analyses were used to identify factors associated with complications and delayed IA occlusion.

**Results:**

The patient age was 55 (47, 59) years, with 37 (52.1%) males. The follow-up duration was 9 (7, 15) months. Postoperative complications included ischemic events (16.9%), hemorrhagic events (4.2%), and asymptomatic in-stent stenosis (12.7%). The 1-year complete occlusion rate was 52%, whereas the long-term occlusion rate gradually increased to 80% with follow-up beyond 12 months. Univariable analysis showed PED length was associated with both delayed IA occlusion [odds ratio (OR) = 0.91, 95% confidence interval (CI) = 0.83–1.00; *p* = 0.040] and ischemic complications (OR = 1.36, 95% CI = 1.11–1.66; *p* = 0.003) and also revealed that pre-existing parent vessel stenosis was strongly associated with asymptomatic in-stent stenosis (OR = 13.50, 95% CI = 2.80–65.04; *p* = 0.001); multivariable analysis confirmed PED length as an independent predictor of ischemic complications (OR = 1.31, 95% CI = 1.06–1.63; *p* = 0.013).

**Conclusion:**

PED deployment in small-caliber parent vessels (<2 mm) is technically feasible and clinically viable, with favorable IA occlusion rates and an acceptable safety profile. These findings support the off-label use of PEDs in this challenging cohort, provided rigorous patient selection, optimal procedural planning and close follow-up are implemented.

## Introduction

Intracranial aneurysm (IA) constitutes a prominent cerebrovascular pathology, with a prevalence of approximately 3–5% in the general population (1). They carry a substantial risk of rupture, which can result in subarachnoid hemorrhage (SAH), associated with a mortality rate of roughly 50% and significant morbidity among survivors (2, 3). Traditional therapeutic strategies, such as microsurgical clipping and endovascular coiling, have long served as the mainstays for IA management; however, these approaches are often constrained in complex cases like wide-necked or fusiform IA, where incomplete IA occlusion, recurrence, and procedural complications remain common challenges (4, 5). The emergence of flow diversion stent (FDS), notably the Pipeline Embolization Device (PED, Medtronic), has ushered in a paradigm shift in endovascular therapy. FDS facilitate endoluminal reconstruction by redirecting hemodynamics, promoting IA thrombosis, and inducing neointimal proliferation over the stent, thereby achieve higher long-term IA occlusion rates compared to conventional endovascular approaches endovascular techniques (6, 7).

Initially approved by the U. S. Food and Drug Administration for large or giant wide-necked IA located in the proximal internal carotid artery (ICA), the PED has increasingly been used off-label for a broader range of IA, including those in distal anterior and posterior circulation segments (8, 9). However, deployment of the PED in small-caliber vessels (mean diameter <2 mm) presents unique challenges, given the device’s nominal minimum diameter of 2.5 mm. This mismatch between vessel and stent diameters raises concerns about potential vessel injury, acute in-stent thrombosis, delayed vessel occlusion, and higher rates of ischemic complications arising from the anatomical fragility and tortuosity of distal vessels, as well as hemorrhagic complications, such as delayed IA rupture and distal parenchymal hemorrhage induced by hemodynamic redirection following PED placement (10, 11). Early studies evaluating FDS use in vessels ≤ 2.5 mm have reported procedural complication rates of 9–10%, symptomatic stroke incidences of approximately 7–8%, and mid-term follow-up complete occlusion rates of 70–75%. These findings suggest efficacy comparable to that in larger vessels but with heightened periprocedural risks, which warrant meticulous patient selection and antiplatelet therapy management (12).

Despite these insights, evidence specifically focusing on PED placement in parent vessels < 2 mm remains scarce. Most existing data are derived from small single-center retrospective series or systematic reviews, underscoring the need for additional research on mid- to long-term safety and angiographic outcomes (13). The present multicenter retrospective study aims to assess the safety and efficacy of PED deployment for IA originating from small parent vessels (< 2 mm) by analyzing clinical and radiological outcomes. This work thereby enhances the evolving understanding of off-label PED use in this challenging IA subset.

## Materials and methods

### Study design

This retrospective multicenter study was approved by the ethics committees of all participating centers and conducted in compliance with the principles of informed consent, with all patients providing written informed consent prior to treatment (Approval Number: KY2025-345-02).

From the prospectively maintained institutional IA databases of three comprehensive stroke centers, we retrospectively identified patients who underwent PED Flex placement, with at least one PED Flex deployed in a parent vessel with a mean diameter <2 mm. Patients with intracranial tumors, cerebrovascular malformations, traumatic or infectious dissections, polycystic kidney diseases, rheumatoid autoimmune diseases, ruptured IA, severe intracranial atherosclerotic stenosis/occlusion or those receiving FDS treatments at other institutions were excluded because these conditions may substantially affect intracranial vascular morphology, hemodynamics, or treatment-related risks. To isolate and accurately evaluate the safety and efficacy of PED for IA, these potential confounding factors were excluded from the study cohort. The study period included cases treated between 2018 and 2022. Treatment decisions were made on a case-by-case basis by a multidisciplinary endovascular team consisting of dual-trained neurosurgeons and neurointervenionalists. The Measurements of proximal and distal parent vessel diameters, aneurysm neck, and maximum IA diameter, were performed based on 3D rotational digital subtraction angiography (DSA) images with standard calibration and mean vessel diameters were computed for each case. All imaging evaluations were independently conducted by two neurosurgeons with more than 5 years of neurointerventional experience. Any discrepancies between the two readers were resolved through consensus discussion. A third senior neurosurgeon with more than 5 years of experience supervised the entire imaging review process and took responsibility for the accuracy and validity of the final measurements. Both anterior and posterior circulation IA meeting the primary inclusion criteria were included. Data collection encompassed relevant patient demographics (age, sex), comorbidities (hypertension, hyperlipidemia, diabetes, coronary artery disease, prior stroke, and history of SAH), IA characteristics (type, location, size and parent vessel diameter), clinical presentation, immediate and delayed complications, and follow-up outcomes (both radiological and clinical).

### Antiplatelet therapy

CYP2C19 genotyping and antiplatelet responsiveness testing were consistently performed across all participating centers. All patients received standard dual antiplatelet therapy (DAPT), consisting of 100 mg aspirin and 75 mg clopidogrel daily, initiated at least 5 days preoperatively and continued for 3–12 months postoperatively. Clopidogrel was switched to ticagrelor (90 mg twice daily) in patients identified as clopidogrel-resistant via CYP2C19 genotyping. For acutely ruptured IA treated emergently, patients received a loading dose of DAPT (300 mg aspirin and 300 mg clopidogrel) preoperatively, followed by an intravenous glycoprotein IIb/IIIa inhibitor (tirofiban; weight-adjusted dosage) administered for 12–24 h. Subsequent maintenance was continued with oral standard DAPT. The duration of antiplatelet therapy was adjusted according to postoperative DSA follow-up findings such as lifelong aspirin administration or discontinuation after a specified duration.

### Treatment strategy

All procedures were performed under general anesthesia. Vascular access was obtained via the right common femoral artery. A guiding catheter was then navigated to the ipsilateral ICA or vertebral artery, based on the IA’s location. Subsequently, a comprehensive evaluation of the parent vessel and IA was conducted to formulate the treatment strategy. Recorded anatomical characteristics included IA type, neck, maximum IA diameter, parent vessel diameter, and planned PED length (with a minimum 3-mm safety margin extending proximally and distally beyond the IA neck). PED deployment was performed using a triaxial system, consisting of a guiding catheter, intermediate catheter, and either a Marksman™ (Medtronic) or Phenom-27™ (Medtronic) microcatheter advanced over a micro-guidewire. Following PED deployment, immediate postprocedural DSA was acquired in magnified views for all patients to confirm adequate device positioning across the IA neck and optimal vessel wall apposition. Additionally, standard angiographic views were obtained to exclude thromboembolic or hemorrhagic complications. Intravenous heparin (50–70 U/kg) was administered after general anesthesia if one or more of the following conditions were present: preoperative platelet function test failing to meet the inhibition standard, emergency patients requiring flow diversion device implantation, prolonged surgical duration, tortuous blood vessels, severe stenosis of the parent vessel, multiple intracranial arterial stenosis, or poor local stent apposition requiring massage to achieve optimal apposition. An additional 1,000 U of heparin was given every 1 h during the operation. The operator could use tirofiban instead of heparin. Regularly, tirofiban was administered by intravenous pump at a rate of 0.1 mL•kg/h during the operation under the same indications as heparin, and the administration was discontinued 12 h after the operation. For patients with severe intraoperative vascular spasm that did not resolve spontaneously after 5–10 min of observation, 0.5 mg of nimodipine was slowly injected intraoperatively. In addition, oral or enteral nimodipine (60 mg every 4 h for 21 consecutive days) was administered to patients with aneurysmal SAH. Hemostasis at the femoral access site was achieved with an arterial closure device.

### Outcomes

The primary clinical safety endpoint included procedure-related death, symptomatic ischemic stroke, asymptomatic in-stent stenosis or occlusion, and procedure-related intracranial hemorrhage. Symptomatic ischemic stroke and procedure-related intracranial hemorrhage were defined as new focal neurological deficits consistent with territorial ischemia or hemorrhage, confirmed by postoperative CT or MRI. In-stent stenosis was defined as any reduction in the contrast-filled lumen of the parent vessel on follow-up DSA. On DSA images, ISS is visualized as a discernible gap between the contrast-opacified vessel lumen and the inner wall of the PED (14). Clinical outcomes were assessed using the modified Rankin Scale (mRS) score, with evaluations performed at discharge and during scheduled follow-up visits (mRS score: 0 = completely asymptomatic, 1 = symptomatic but without significant functional impairment, able to perform all daily activities and work, 2 = mild disability, unable to perform all activities but can manage personal affairs without assistance, 3 = moderate disability, requiring some help but able to walk independently, 4 = moderately severe disability, unable to walk independently, requiring assistance in daily life, 5 = severe disability, bedridden, incontinent, completely dependent on others for daily activities, 6 = death). The primary efficacy endpoint was complete occlusion of the target IA, as determined by the O’Kelly-Marotta (OKM) grading scale, on follow-up DSA. 1-year aneurysm occlusion rate was defined as complete aneurysm occlusion (OKM class D) documented on the patient’s DSA within the 12-month follow-up. Final aneurysm occlusion rate was defined as complete aneurysm occlusion (OKM class D) documented on the patient’s last available DSA follow-up. Delayed aneurysm occlusion was defined as persistent contrast filling within the aneurysm sac on DSA performed at ≥12 months after PED implantation.

### Statistical analysis

Statistical analyses were performed using SPSS software (version 25.0; IBM Corp., Armonk, NY, United States). Normally distributed continuous variables were expressed as mean ± standard deviation (SD). Non-normally distributed continuous variables were reported as median (interquartile range, IQR; Q1–Q3). Categorical variables were presented as counts (percentages). Continuous variables were compared using the two-tailed Student’ s *t*-test or Mann–Whitney U test, as appropriate based on data distribution. Categorical variables were compared using the chi-square test or Fisher’ s exact test. Variables with *p* < 0.05 in univariable analysis were included in multivariable logistic regression models to identify independent predictors of procedure-related complications and clinical efficacy outcomes, after adjusting for potential confounding factors. Besides, based on our experience, we estimate that the postoperative complication rate will not exceed 20% of the total number of patients. According to the widely accepted events-per-variable rule (≥10 events per variable), the ideal number of variables for the multivariable model was calculated as approximately 1.5. Therefore, we limited the number of variables included in the multivariable model to a maximum of 2 to maintain statistical reliability. A two-sided *p* < 0.05 was considered statistically significant.

## Results

### Patient demographics and baseline characteristics

**Figure 1:**
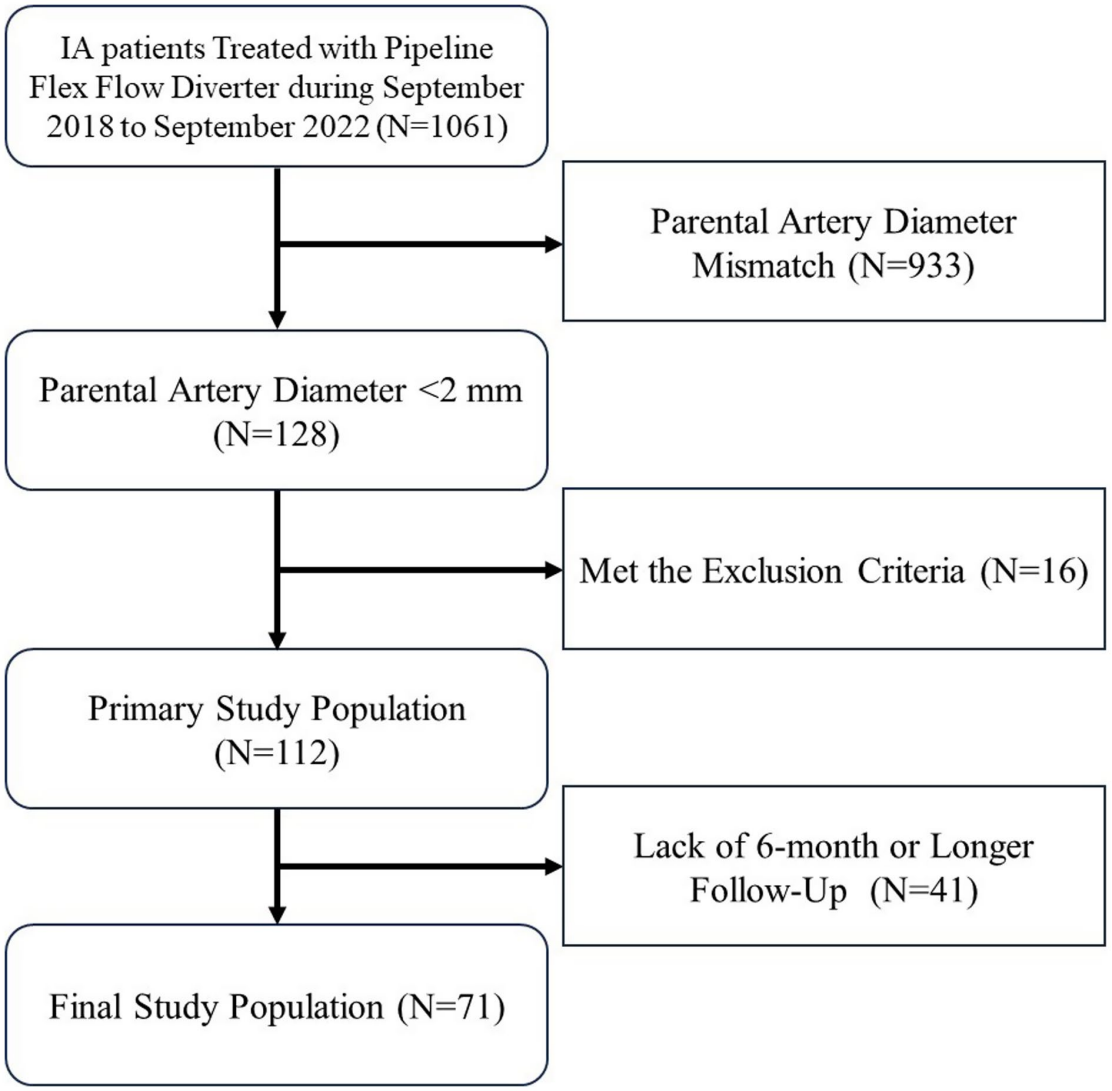
The flowchart of this study. After screening, a total of 71 eligible patients were enrolled in this study. IA, Intracranial aneurysm.

After initial screening, a total of 71 eligible patients were enrolled in this study. Figure 1 illustrates the study flowchart. The patient age was 55 (47, 59) years, with 37 patients (52.1%) being male. Baseline comorbidities included hypertension in 34 patients (47.9%), diabetes in 7 (9.9%), dyslipidemia in 6 (8.5%), coronary artery disease in 8 (11.3%), prior SAH in 11 (15.5%), and prior ischemic stroke in 6 (8.5%). Sixteen patients (22.5%) reported current smoking, and 12 (16.9%) reported regular alcohol consumption. Preprocedural modified Rankin Scale (mRS) scores were 0 in 48 patients (67.6%), 1 in 18 (25.4%), 2 in 2 (2.8%), and 3 in 3 (4.2%). Ten patients (14.1%) had a history of prior endovascular treatment for IA (Table 1).

**Table 1:** Baseline characteristics of the participants.

Variables	Number (proportion)/median (Q1, Q3)
Age	55 (47, 59)
Male	37 (52.1%)
Hypertension	34 (47.9%)
Diabetes	7 (9.9%)
Dyslipidemia	6 (8.5%)
Coronary artery disease	8 (11.3%)
History of SAH	11 (15.5%)
History ischemic stroke	6 (8.5%)
Current smoking	16 (22.5%)
Regular alcohol consumption	12 (16.9%)
Pre-operation mRS	
0	48 (67.6%)
1	18 (25.4%)
2	2 (2.8%)
3	3 (4.3%)
History of endovascular treatment for IA	10 (14.1%)

IA, Intracranial Aneurysm; SAH, subarachnoid hemorrhage; mRS, modified Rankin Scale score: 0 = completely asymptomatic, 1 = symptomatic but without significant functional impairment, able to perform all daily activities and work, 2 = mild disability, unable to perform all activities but can manage personal affairs without assistance, 3 = moderate disability, requiring some help but able to walk independently, 4 = moderately severe disability, unable to walk independently, requiring assistance in daily life, 5 = severe disability, bedridden, incontinent, completely dependent on others for daily activities, 6 = death.

### Procedural and IA characteristics

IA characteristics included bifurcation IA in 39 patients (54.9%), IA with daughter sacs in 12 (16.9%), non-saccular IA in 34 (47.9%), symptomatic IA in 36 (50.7%), ruptured IA in 5 (7.0%) and multiple IA in 15 (21.1%). The IA neck was 6.8 (5.00, 9.79) mm, maximum IA diameter was 10.94 (6.93, 13.6) mm, and parent vessel diameter was 1.8 (1.62, 1.9) mm. Parent vessel stenosis was observed in 14 patients (19.7%) and 4 (5.6%) patients had intracranial arterial stenosis in vessels other than the parent artery. IA locations were as follows: A2 segment in 6 patients (8.5%), A3 segment in 14 (19.7%), M1-M2 segments in 33 (46.5%), M2-M3 segments in 8 (11.3%), P2 segments in 9 (12.7%), and P3 segment in 1 (1.4%). The PED length was 20 (20, 30) mm, PED diameter was 3 (2.5, 3.25) mm, and median diameter mismatch between the PED and parent vessel was 1.2 (0.92, 1.71) mm. Procedural details included deployment of multiple PEDs in 7 patients (9.9%), intraoperative balloon angioplasty in 11 (15.5%), and PED-assisted coiling in 16 (22.5%). Intraoperative medications administered included nimodipine in 17 patients (23.9%), heparin in 32 (45.1%), and tirofiban in 37 (52.1%) (Table 2).

**Table 2:** Procedure-related and follow-up characteristics of the participants.

Variables	Number (proportion)/median (Q1, Q3)
Multiple PED deployment	7 (9.9%)
Intraoperative balloon angioplasty	11 (15.5%)
PED assisted with coils	16 (22.5%)
Nimodipine utilization	17 (23.9%)
Heparin utilization	32 (45.1%)
Tirofiban utilization	37 (52.1%)
Ischemic complication	12 (16.9%)
Procedure-related intracranial hemorrhage	3 (4.2%)
Asymptomatic in-stent stenosis	9 (12.7%)
Delayed occlusion of aneurysm	16 (22.5%)
Procedure-related death	2 (2.8%)
Bifurcation IA	39 (54.9%)
IA with daughter sac	12 (16.9%)
Non-saccular IA	34 (47.9%)
Symptomatic IA	36 (50.7%)
Ruptured IA	5 (7.0%)
Multiple IAs	15 (21.1%)
IA neck (mm)	6.8 (5.00, 9.79)
Maximum diameter of the IA (mm)	10.94 (6.93, 13.60)
Mean diameter of parent vessel (mm)	1.8 (1.62, 1.9)
Stenosis of parent vessel	14 (19.7%)
Stenosis of other intracranial arteries	4 (5.6%)
IA Location	
A2	6 (8.5%)
A3	14 (19.7%)
M1-M2	33 (46.5%)
M2-M3	8 (11.3%)
P2	9 (12.7%)
P3	1 (1.4%)
Lenth of PED (mm)	20 (20, 30)
Diameter of PED (mm)	3 (2.5, 3.25)
Diameter difference between PED and parent vessel (mm)	1.2 (0.92, 1.71)
PED Covered ≥ 2 branches observed in DSA	55 (77.5%)
Incomplete wall apposition	5 (7.0%)
Duration of aspirin use post-operation (month)	12 (6, 24)
Duration of clopidogrel use post-operation (month)	6 (3, 6)
Clopidogrel replaced by ticagrelor	2 (2.8%)
Last follow-up time (month)	9 (7, 15)
Last follow-up mRS	
0	56 (78.9%)
1	7 (9.9%)
2	3 (4.2%)
3	2 (2.8%)
4	1 (1.4%)

IA, Intracranial Aneurysm; PED, Pipeline Embolization Device; mRS, modified Rankin Scale score: 0 = completely asymptomatic, 1 = symptomatic but without significant functional impairment, able to perform all daily activities and work, 2 = mild disability, unable to perform all activities but can manage personal affairs without assistance, 3 = moderate disability, requiring some help but able to walk independently, 4 = moderately severe disability, unable to walk independently, requiring assistance in daily life, 5 = severe disability, bedridden, incontinent, completely dependent on others for daily activities, 6 = death; A2, A3: segment of the anterior cerebral artery; M1, 2, M3: segment of the middle cerebral artery; P: P2, P3: segment of the posterior cerebral artery.

### Post-operative and follow-up outcomes

**Figure 2:**
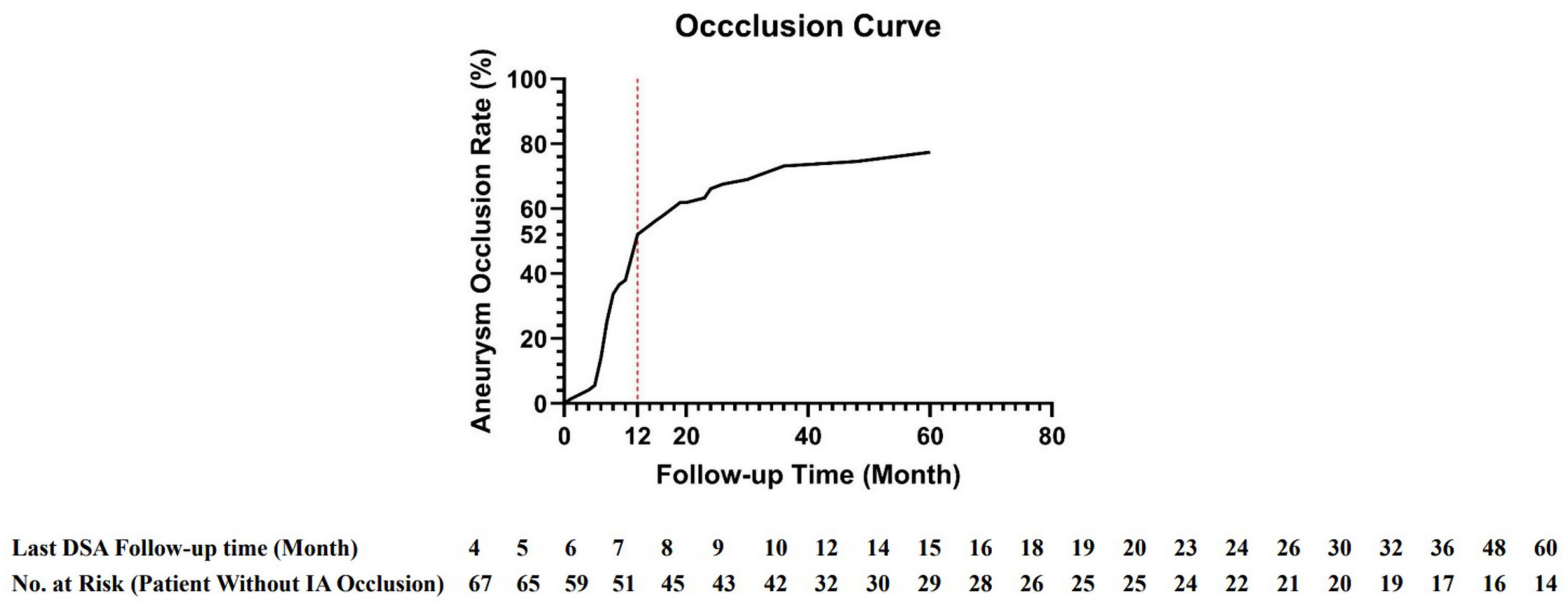
The IA occlusion curve for PED deployed in small caliber vessels (< 2 mm) for IA treatment. This curve indicates that more than 50% of patients achieve IA occlusion within 1 year after PED deployment. As follow-up time extends, an increasing number of patients attain satisfactory efficacy outcomes, underscoring the clinical significance of long-term follow-up for such patients.

Postoperative complications included ischemic events in 12 patients (16.9%), procedure-related intracranial hemorrhage events in 3 (4.2%), asymptomatic in-stent stenosis in 9 (12.7%), delayed IA occlusion in 16 (22.5%), and procedure-related death in 2 (2.8%). Both patients suffered procedure-related death succumbed to fatal intracranial hemorrhage that occurred in the early postoperative period following PED implantation. The clinical progression of both cases was rapid, and thus neither patient underwent postoperative DSA evaluation. Based on the findings of emergency postoperative CT, we hypothesize that both events were attributable to delayed IA rupture after the PED procedure. The follow-up duration was 9 (7, 15) months, with a maximum follow-up duration of 60 months. Durations of postoperative antiplatelet therapy were 12 (6, 24) months for aspirin and 6 (3, 6) months for clopidogrel; clopidogrel was replaced with ticagrelor in 2 patients (2.8%). At the final DSA follow-up, the primary efficacy endpoint (OKM grade D, complete occlusion) was achieved in 80% of cases. For one-year clinical outcomes, assessed using the mRS: 56 patients (78.9%) achieved 0 score, 12 (16.9%) had 1–3 scores, 1 (1.4%) had 4 scores (Table 2). The IA occlusion curve (Figure 2) depicts the cumulative IA occlusion rate over time, showing a rapid increase within the first 12 months, followed by a plateau phase extending to 60 months, with the rate stabilizing at approximately 80%. This trend highlights the time-dependent efficacy of PED in small-caliber vessels, with the majority of IA occlusions occurring within the initial year. Detailed data on the number of patients at each follow-up time point and the DSA completion rate at each time point are provided in Supplementary Table 1.

### Factors associated with post-operative complications and delayed IA occlusion

To avoid collinearity, we excluded variables with variance inflation factor >10 from the logistic regression analysis. Univariable logistic regression analysis showed that IA neck [odds ratio (OR) = 1.10, 95% confidence interval (CI) = 1.00–1.21; *p* = 0.039] and PED length (OR = 1.36, 95% CI = 1.11–1.66; *p* = 0.003) were significantly associated with postoperative ischemic complications. In multivariable logistic regression analysis, PED length remained an independent predictor of ischemic complications (OR = 1.31, 95% CI = 1.06–1.63; *p* = 0.013) (Table 3). For delayed IA occlusion, univariable analysis revealed that PED length was a significant associated factor (OR = 0.91, 95% CI = 0.83–1.00; *p* = 0.040). Regarding asymptomatic in-stent stenosis, parent vessel stenosis was strongly associated with this complication (OR = 13.50, 95% CI = 2.80–65.04; *p* = 0.001). Given that only one variable reached statistical significance in the univariable analysis for delayed IA occlusion or asymptomatic in-stent stenosis, multivariable analysis was not deemed appropriate to avoid overfitting (Table 4).

**Table 3:** Binary logistic regression of factors associated with post-operative ischemic complications.

Characteristics	Univariable				Adjusted			
	*P*	OR	95%CI		*P*	OR	95%CI	
Female	0.493	0.67	0.21	2.12	/	/	/	/
Age	0.815	1.00	0.97	1.04	/	/	/	/
Hypertension	0.915	0.94	0.3	2.94	/	/	/	/
Diabetes	0.991	NA	NA	NA	/	/	/	/
Dyslipidemia	0.992	NA	NA	NA	/	/	/	/
Coronary artery disease	0.533	0.50	0.06	4.41	/	/	/	/
History of ischemic stroke	0.451	2.00	0.33	12.13	/	/	/	/
Current smoking	0.791	0.83	0.20	3.38	/	/	/	/
Regular alcohol consumption	0.679	0.71	0.14	3.64	/	/	/	/
IA location								
A2	ref	ref	ref	ref	/	/	/	/
A3	0.992	NA	NA	NA	/	/	/	/
M1-M2	0.279	0.37	0.06	2.21	/	/	/	/
M3	0.148	0.14	0.01	1.99	/	/	/	/
P1-P2	0.12	0.12	0.01	1.72	/	/	/	/
P3	0.998	NA	NA	NA	/	/	/	/
Bifurcation IA	0.657	1.30	0.41	4.14	/	/	/	/
Non-saccular IA	0.635	1.32	0.42	4.13	/	/	/	/
IA with daughter sac	0264	2.18	0.56	8.56				
Stenosis of parent vessel	0.992	NA	NA	NA	/	/	/	/
Multiple PED deployment	0.155	3.25	0.64	16.48	/	/	/	/
PED covered ≥ 2 branches observed in DSA	0.823	0.848	0.20	3.594	/	/	/	/
Incomplete wall apposition	0.999	NA	NA	NA	/	/	/	/
PED with coils	0.266	2.05	0.58	7.21	/	/	/	/
Intraoperative balloon angioplasty	0.589	1.50	0.34	6.52	/	/	/	/
Multiple IAs	0.992	NA	NA	NA	/	/	/	/
Nimodipine utilization	0.688	0.75	0.18	3.05	/	/	/	/
Heparin utilization	0.889	1.09	0.35	3.40	/	/	/	/
Tirofiban utilization	0.21	2.15	0.65	7.09	/	/	/	/
Mean diameter of parent vessel	0.957	0.93	0.07	13.08	/	/	/	/
Maximum diameter of IA	0.07	1.11	0.99	1.24	/	/	/	/
IA neck	**0.039**	1.10	1.00	1.21	0.414	1.05	0.94	1.17
Diameter of PED	0.2	1.96	0.70	5.46	/	/	/	/
Length of PED	**0.003**	1.36	1.11	1.66	**0.013**	1.31	1.06	1.63
Duration of aspirin use post-operation	0.172	1.05	0.98	1.13	/	/	/	/
Duration of clopidogrel use post-operation	0.179	0.81	0.59	1.10	/	/	/	/

IA, Intracranial Aneurysm; PED, Pipeline Embolization Device; A2, A3: segment of the anterior cerebral artery; M1, 2, M3: segment of the middle cerebral artery; P: P2, P3: segment of the posterior cerebral artery; NA: Not Applicable due to low event frequency. Bold values indicates *p*-value of less than 0.05.

**Table 4:** Binary logistic regression of factors associated with delayed IA occlusion and asymptomatic in-stent stenosis.

Characteristics	Delayed IA occlusion				Asymptomatic in-stent stenosis			
	Univariable				Univariable			
	*P*	OR	95%CI		*P*	OR	95%CI	
Female	0.449	0.65	0.21	1.99	0.623	1.42	0.35	5.80
Age	0.172	0.97	0.92	1.01	0.346	0.98	0.94	1.02
Hypertension	0.707	1.24	0.40	3.80	0.623	1.42	0.35	5.80
Diabetes	0.587	1.84	0.20	16.49	0.893	1.17	0.12	10.99
Dyslipidemia	0.992	NA	NA	NA	0.76	1.42	0.15	13.81
Coronary artery disease	0.991	NA	NA	NA	0.281	2.67	0.45	15.86
History of ischemic stroke	0.721	1.50	0.16	13.86	0.992	NA	NA	NA
Current smoking	0.789	0.84	0.23	3.07	0.981	0.98	0.18	5.26
Regular alcohol consumption	0.596	1.56	0.30	7.96	0.65	1.49	0.27	8.22
IA location								
A2	ref	ref	ref	ref	ref	ref	ref	ref
A3	0.992	NA	NA	NA	0.995	NA	NA	NA
M1-M2	0.992	NA	NA	NA	0.996	NA	NA	NA
M3	0.992	NA	NA	NA	0.995	NA	NA	NA
P1-P2	0.992	NA	NA	NA	0.995	NA	NA	NA
P3	0.993	NA	NA	NA	1	1.00	NA	NA
Bifurcation IA	0.491	0.67	0.21	2.10	0.176	0.36	0.08	1.58
Non-saccular IA	0.064	0.33	0.10	1.07	0.623	1.42	0.35	5.80
IA with daughter sac	0.332	0.51	0.13	1.98	0.624	0.58	0.07	5.12
Stenosis of parent vessel	0.416	1.95	0.39	9.81	**0.001**	13.5	2.80	65.04
Multiple PED deployment	0.991	NA	NA	NA	0.203	3.26	0.53	20.06
PED with coils	0.681	1.34	0.33	5.44	0.992	NA	NA	NA
Intraoperative balloon angioplasty	0.269	3.33	0.39	28.24	0.129	3.37	0.70	16.26
PED covered ≥ 2 branches observed in DSA	0.109	5.63	0.68	46.37	0.412	0.53	0.18	2.41
Incomplete wall apposition	0.346	2.48	0.38	16.29	0.614	1.81	0.18	18.31
Multiple IAs	0.266	0.49	0.14	1.72	0.931	1.08	0.2	5.81
Mean diameter of parent vessel	0.556	2.12	0.17	25.92	0.303	0.21	0.01	4.10
Maximum diameter of IA	0.453	0.96	0.86	1.07	0.759	1.02	0.89	1.17
IA neck	0.543	0.95	0.81	1.12	0.176	0.84	0.66	1.08
Diameter of PED	0.874	0.92	0.32	2.62	0.07	0.13	0.02	1.17
Length of PED	**0.040**	0.91	0.83	1.00	0.583	0.96	0.85	1.10
Duration of aspirin use post-operation	0.989	1.00	0.93	1.07	0.66	0.98	0.90	1.07
Duration of clopidogrel use post-operation	0.692	0.95	0.74	1.22	0.62	1.08	0.80	1.47

IA, Intracranial Aneurysm; PED, Pipeline Embolization Device; A2, A3, segment of the anterior cerebral artery; M1, 2, M3: segment of the middle cerebral artery; P: P2, P3: segment of the posterior cerebral artery; NA, Not Applicable due to low event frequency.

### Illustrative cases

**Figure 3:**
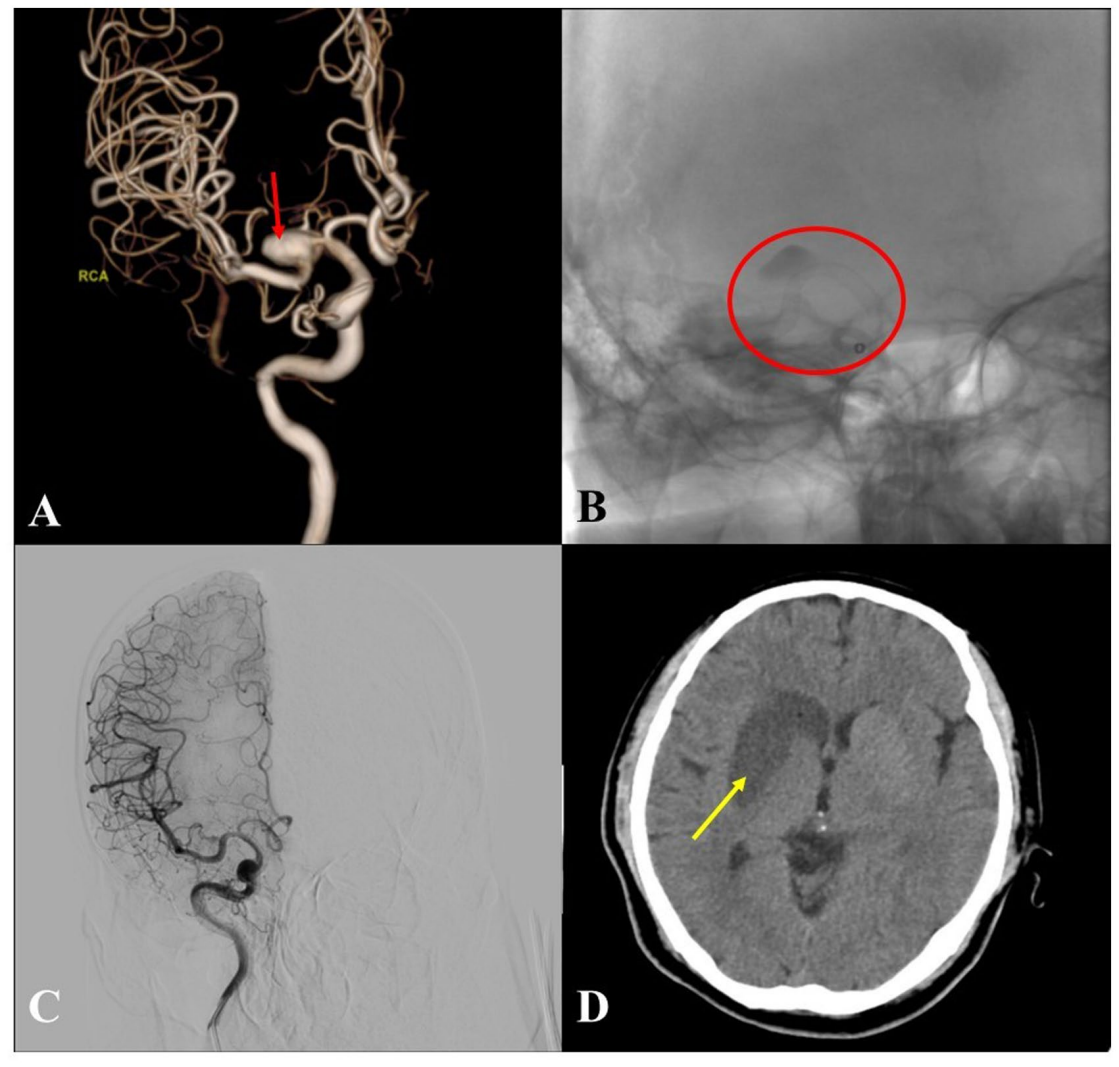
A case of postoperative cerebral infarction after PED implantation. **(A)** Intraoperative 3D angiography revealed a non-saccular IA in the M1 segment of the right middle cerebral artery (red arrow). **(B)** A 3.25 × 25 mm PED Flex was placed in the right C7–M1 segment, and marked remnant of contrast within the IA was observed (red circle). **(C)** On the second day after intervention, the patient developed weakness in the left side of the body. Follow-up DSA revealed that the IA had almost completely disappeared, and the right middle cerebral artery remained patent; immediately intravenous infusion therapy with Tirofiban was initiated. **(D)** A follow-up head CT scan on the third day after intervention revealed a cerebral infarction in the right basal ganglia region (yellow arrow).

**Figure 4:**
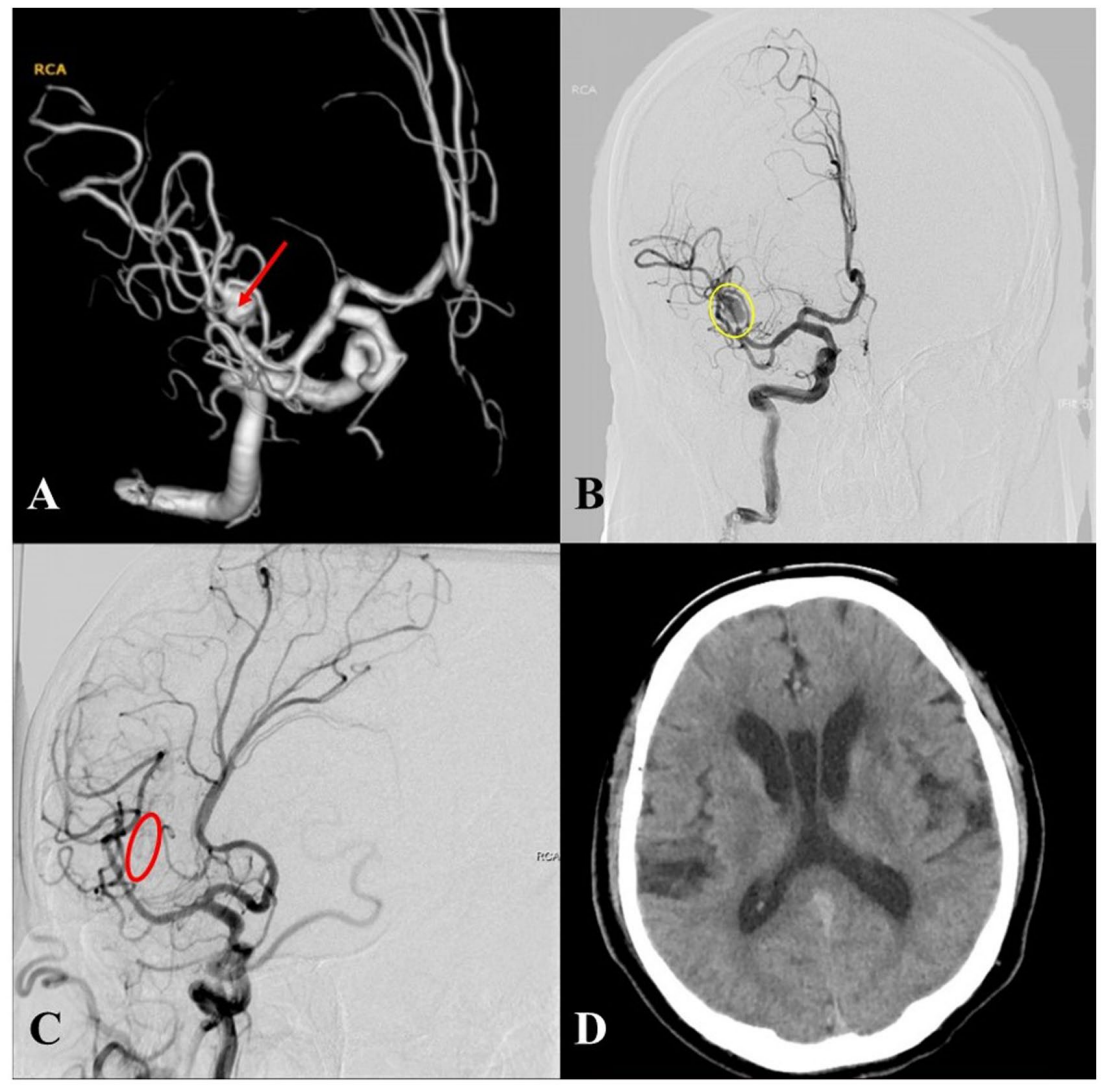
A case of postoperative chronic parent vessel occlusion after PED implantation. **(A)** Intraoperative 3D angiography revealed a non-saccular IA in the M3 segment of the right middle cerebral artery (red arrow). **(B)** A 3.00 × 20 mm PED Flex was placed in the right M2–M3 segment, marked remnant of contrast within the IA was observed in the immediate postoperative angiography (yellow circle). **(C)** One-year postoperative follow-up DSA revealed occlusion of the parent vessel (red circle), with the patient reporting no discomfort after intervention. **(D)** One-year follow-up head CT scan showed no evidence of cerebral infarction at the site of the occluded artery.

To illustrate the procedure-related complications observed in this cohort, two representative cases are presented. A patient with a non-saccular IA of the right M1 segment was treated with a 3.25 × 25 mm PED Flex. On post-procedural day 2, the patient developed left-sided limb weakness. Urgent DSA revealed near-complete IA occlusion with a patent middle cerebral artery; the patient was promptly treated with tirofiban infusion. A head CT scan obtained on post-procedural day 3 confirmed an acute infarction in the right basal ganglia. During subsequent follow-up, the patient’s limb weakness showed no significant improvement (Figure 3). Another patient with a non-saccular IA of the right M3 segment was managed with a 3.0 × 20 mm PED Flex. At the 1-year follow-up, DSA demonstrated asymptomatic parent vessel occlusion, and a head CT scan showed no evidence of cerebral infarction at target area (Figure 4).

## Discussion

The clinical use of FDS has expanded considerably in recent years, primarily due to their proven efficacy and favorable safety profile (15–17). Although previous studies have reported off-label PED application in distal non-Circle of Willis territories with promising preliminary outcomes, most lack specific focus on target vessel caliber, particularly vessels <2 mm (11). The present study specifically evaluated the procedural safety and efficacy of PED deployment for IAs arising from small-caliber parent vessels (<2 mm), further supporting the device’ s applicability in off-label distal vascular segments. Published prospective multicenter cohort studies and systematic reviews have demonstrated that PED treatment for IA in proximal, larger caliber vessels achieves a long-term complete occlusion rate of 86–95%, with ischemic and hemorrhagic complication rates of approximately 8 and 3%, respectively (18–25). In the present study, which focused on IA arising from small-caliber parent vessels (< 2 mm), a population representing off-label use of PED, the long-term occlusion rate reached 80%, with an ischemic complication rate of 16.9% and procedure-related intracranial hemorrhage rate of 4.2%. Given the technically challenging anatomy and off-label indication in the present cohort, we believe the deployment of PEDs in small caliber parent vessels (<2 mm) is technically feasible with favorable IA occlusion rate and an acceptable safety profile, despite an elevated ischemic complication rate compared to PED use in proximal, larger vessels. It indicates that PED may be a viable therapeutic option for this challenging IA subset.

A primary concern in treating IA in small arteries is the heightened risk of procedure-related complications, particularly ischemic complications, which constitute a leading cause of morbidity in neurointerventional procedures (12, 25). To contextualize the ischemic complication rate observed in our small-caliber vessel cohort relative to that in proximal territories, we compared our findings with a comprehensive meta-analysis evaluating FDS for predominantly proximal IA (16). That meta-analysis, encompassing 29 studies and 1,524 patients, reported a composite ischemic complication rate of 9.2%, nearly half the incidence observed in our study (16.9%). In contrast, Bender et al. described a similar series focusing on PED treatment of IA in vessels <2.0 mm, reporting three major ischemic strokes (4.5% ischemic complication rate); all were attributed to acute stent occlusion detected intraoperatively or within 24 h post-implantation (26). Maintaining heightened vigilance for such complications is paramount in small-caliber vessels, where reduced blood flow velocities and higher metal-to-lumen ratios may theoretically augment the risk of in-stent occlusion. Our findings suggest that neurointerventionalists should conduct a more thorough risk assessment and carefully consider alternative therapeutic approaches when deploying PEDs in vessels <2 mm. Notably, however, no comparative studies to date have definitively confirmed a higher complication incidence in small-caliber vessels (13), highlighting the need for larger, well-designed studies to clarify this association.

Another key concern in treating IA in small distal vessels is the potential coverage of eloquent side branches, which may increase the risk of branch occlusion. Bender et al., who reported favorable outcomes with PEDs for IA in distal vessels <2.0 mm, also documented instances of acute in-stent thrombosis, successfully managed with intraoperative pharmacological intervention (26). Such events are infrequent, with reported rates of 2–5%, and rarely result in neurological deficits from ischemic lesions (27). The mechanism of FDS involves disrupting intra-aneurysmal blood flow while preserving physiological perfusion in the parent vessel and covered branches via pressure gradients. Side-branch occlusion is thus attributed to diminished pressure gradients, particularly in vascular territories with robust collateral circulation (6, 28). Notwithstanding, one case in our study (Figure 3) demonstrated that side-branch occlusion can be symptomatic; notably, the affected branch arose directly from the IA dome rather than the parent vessel, suggesting unique angioarchitecture and hemodynamic characteristics. However, in the present study, we investigate PED coverage of ≥2 visible branch vessels, and incorporated this variable into statistical analyses and no significant associations being detected between this variable and postoperative ischemic complications or delayed aneurysm occlusion. The lack of significant findings may be due to the relatively small sample size, which limited the statistical power to identify subtle associations. Besides, systematic investigations into flow patterns across diverse angioarchitectural configurations remain lacking. Furthermore, no consensus has been reached regarding whether assisted coil embolization should be performed when branching vessels originate within the IA itself.

A meta-analysis focusing on distal anterior circulation IA reported a long-term satisfactory occlusion rate of approximately 83% at a mean 12-month follow-up (12). In comparison, our study achieved the primary efficacy endpoint (complete occlusion) in 52% of cases at 1-year follow-up, and with extended follow-up, the occlusion rate in our cohort increased to 80%. Notably, our logistic regression analysis revealed that PED length was associated with both delayed IA occlusion and postoperative ischemic complications. Deploying longer PEDs in small-caliber vessels increases the metal surface area exposed to blood flow and raises the likelihood of covering more side branches, which may contribute to higher ischemic risks. On the other hand, longer PEDs may reduce technical complexity for operators, as they facilitate adequate coverage of the IA neck and ensure optimal stent-to-vessel wall apposition. This improved apposition potentially enhances hemodynamic redirection and accelerates neointimal hyperplasia, which may explain the association between longer PEDs and delayed IA occlusion. Based on our analysis showing that longer PEDs are independently associated with an increased risk of ischemic complications, we recommend using the shortest effective PED length sufficient to cover the aneurysm neck and ensure secure landing zones when treating IA in small-caliber vessels (<2 mm). This strategy may help minimize excessive metal surface exposure, reduce the likelihood of side-branch coverage, and lower the risk of thromboembolic events.

However, the implications of FDS oversizing merit attention. The braided design of the PED results in device elongation when oversized, which complicates accurate prediction of the distal and proximal landing zone. Moreover, metal coverage typically exhibits a parabolic relationship with the vessel-to-stent diameter deference; oversizing beyond a certain threshold generally reduces metal coverage, potentially delaying neointimal hyperplasia and IA occlusion (29, 30). Besides, reduced metal coverage may mitigate the risk to jailed side branches. Although several studies have explored the effects of FDS oversizing on covered branches, the precise clinical implications remain controversial and may vary by anatomical location (31). In the present study, univariable and multivariable analyses revealed no association between stent size and delayed IA occlusion. Thus, the aforementioned theoretical relationship between oversizing, metal coverage, and outcomes may require further validation in larger-scale controlled studies. Furthermore, technical complications (e.g., vessel dissection, guidewire perforation) are theoretically more prevalent in small-caliber vessels. Notably, no such events were observed in our cohort, and previous studies have reported that these complications typically exert minimal clinical impact (32).

## Limitations

This study has inherent limitations due to its retrospective, observational, and single-arm design. Data were extracted from operator-reported clinical records, which may introduce potential reporting bias. Second, certain variables exhibited heterogeneity, such as the use of adjunctive coiling, follow-up protocols, and pharmacological regimens, attributable to variations in clinical practices across participating centers. Third, this study only reports intermediate-term follow-up results, which limits the generalizability of long-term efficacy and safety outcomes. Finally, due to the small sample size and retrospective nature, which could lead to underreporting of rare events such as incomplete wall apposition for PED, which may recognize as potential contributor to peri-procedural ischemic complications in PED treatment.

## Conclusion

The deployment of PEDs in small-caliber parent vessels (<2 mm) is technically feasible and clinically viable, with favorable IA occlusion rates (up to 80%) and an acceptable safety profile in our cohort, despite an elevated ischemic complication rate compared to PED use in proximal, larger vessels. Vigilance regarding procedure-related complications specific to small-caliber vessels (e.g., ischemic events, side-branch occlusion) is paramount to enable early detection and intervention, thereby minimizing potential neurological morbidity.

## Data Availability

The raw data supporting the conclusions of this article will be made available by the authors, without undue reservation.
