# Supplementary material for: Multicenter experience of pipeline embolization device used in small caliber vessels (< 2 mm) for intracranial aneurysm treatment and mid-term results
Source: Front Neurol. 2026 Apr 8;17:1778770. doi: 10.3389/fneur.2026.1778770 (PMC13099330; doi:10.3389/fneur.2026.1778770)
Supplement: Supplementary file 1 [file Table_1.DOCX]

Supplemental Table 1. Time and Number of Patient Received Last DSA Follow-Up and DSA Follow-Up Completion Rate

| Last DSA Follow-up time (Month) | 4 | 5 | 6 | 7 | 8 | 9 | 10 | 12 | 14 | 15 | 16 | 18 | 19 | 20 | 23 | 24 | 26 | 30 | 32 | 36 | 48 | 60 |
| --- | --- | --- | --- | --- | --- | --- | --- | --- | --- | --- | --- | --- | --- | --- | --- | --- | --- | --- | --- | --- | --- | --- |
| No. of Patient Received Last DSA Follow-Up | 3 | 2 | 9 | 9 | 7 | 4 | 2 | 13 | 2 | 1 | 1 | 2 | 1 | 1 | 1 | 3 | 1 | 1 | 1 | 2 | 1 | 2 |
| Cumulative No. of Patient Received Last DSA Follow-Up | 3 | 5 | 14 | 23 | 30 | 34 | 36 | 49 | 51 | 52 | 53 | 55 | 56 | 57 | 58 | 61 | 62 | 63 | 64 | 66 | 67 | 69 |
| DSA Completion Rate | 4.2% | 7.0% | 19.7% | 32.4% | 42.3% | 47.9% | 50.7% | 69.0% | 71.8% | 73.2% | 74.6% | 77.5% | 78.9% | 80.3% | 81.7% | 85.9% | 87.3% | 88.7% | 90.1% | 93.0% | 94.4% | 97.2% |

DSA, digital subtraction angiography.
